# Naming disease, shaping care: patient perspectives matter

**DOI:** 10.1016/j.eclinm.2026.104125

**Published:** 2026-07-24

**Authors:** eClinicalMedicine

The recent renaming of polycystic ovary syndrome (PCOS) to polyendocrine metabolic ovarian syndrome (PMOS) marks more than a change in terminology. Nomenclature guides research agendas, influences diagnosis, shapes societal perceptions, and can either facilitate or impede access to care. For this reason, medical terminology is increasingly the subject of negotiation between clinicians, researchers, and the patients who live with these conditions every day. More broadly, increased patient and public involvement in health-care research and delivery has gained international momentum over the past two decades. As well as being ethically sound, meaningful patient involvement improves the quality of research outputs and patient outcomes by providing an additional source of knowledge.

As outlined in the international consensus process published in *The Lancet*, the change from PCOS to PMOS improves alignment with clinical features and has been described as an evolution based on scientific understanding. The new name seeks to reduce stigma and improve recognition as an endocrine condition with metabolic, reproductive, psychological, and dermatological implications. Given the high prevalence of PMOS (7–12% globally), and against a backdrop of systematic failures in diagnosis and management (with up to 70% of affected women undiagnosed), it is hoped that this shift will improve care for patients. The new PMOS terminology emerged from a 14-year process involving 56 professional and patient organisations, Delphi consensus exercises, and substantial involvement of people with lived experience (PWLE). Scientific accuracy, reduction of stigma, and feasibility of implementation were prioritised throughout. This effort followed a multistakeholder, international consultation, published in *eClinicalMedicine*, to explore perspectives on PCOS, priority research gaps, and potential renaming. With involvement from patients (via Verity [UK], PCOS Challenge [USA], and the PCOS Association of Australia) and health professionals across six continents, this initiative established a mandate for change.

The PMOS transition provides a timely case study of a shift to greater recognition of patient perspectives. PWLE and their representatives were meaningfully involved in co-creation. Patients shaped governance, co-led on survey design and workshops, and had real voting power (patients voted 3:1 versus health professionals). Patients were no longer passive observers of diagnostic language but active contributors in their own experiences in a way that complements, and completes, the scientific evidence. As patients experience the downstream effects of disease nomenclature in health-care systems and society, their perspectives offer invaluable insights into the emotional and personal repercussions of these labels, as well as whether a name promotes understanding, legitimises illness, or contributes to stigma and exclusion.

The PMOS process has been referred to as the most comprehensive medical renaming process ever undertaken, and could act as an exemplar for how to involve a multidisciplinary and diverse set of individuals in making such change, but this is not an isolated example. Long COVID, a term originally coined by patients, offers a tangible example of PWLE leading the way on nomenclature. This grassroots movement accelerated recognition and research. The term rapidly entered public discourse and was subsequently adopted by researchers, clinicians, policymakers, and the media. WHO may have settled on post-COVID-19 condition but long COVID has remained in the lexicon, particularly among patients who have described the importance of a recognisable name. The renaming of non-alcoholic fatty liver disease (NAFLD) to metabolic dysfunction-associated steatotic liver disease (MASLD) also involved patient voice. This name change was made to remove the diagnosis of exclusion (via non-alcoholic) and, similarly to PMOS, improve alignment with the underlying pathogenetic pathways and alleviate potential stigma. A modified Delphi process was led by three large pan-national liver associations, with a dedicated working group on the patient-centred perspective. There is ongoing debate surrounding the use of other terms, such as schizophrenia, which has changed in several Eastern cultures. For example, to *Togo Shitcho Sho* (integration disorder) in Japan and to *Johyeonbyung* (attunement disorder) in South Korea. Elsewhere, surveys in the USA and Italy have shown support for alternative terms, with Altered Perception Syndrome suggested by PWLE in the US study. True and meaningful patient involvement is needed as part of these continued conversations.

Of course, changing the name of a condition is not the end of the story. Adoption by clinicians, patients, and policy makers can face a myriad of challenges, such as diagnostic confusion, loss of historical progress, and operational challenges, and the true value of this change lies in whether it positively impacts clinical behaviour and patient experience. For PMOS, a 3-year implementation process with multilingual resources is planned to support integration across education, electronic health records, and disease classification; culminating in integration into international guidelines in 2028. There are unanswered questions on adoption across languages and cultures, how PCOS and PMOS will coexist, how health literacy around the condition will be affected, and whether the high degree of patient co-development translates to successful adoption is yet to be seen. Collaboration and monitoring of implementation in primary care and across specific groups, such as adolescents with their unique needs and challenges, is crucial to equitable progress.

As healthcare increasingly embraces partnership models of care between healthcare professionals and patients, disease nomenclature should evolve accordingly. The question is no longer simply what diseases are called, but who participates in deciding. PMOS, MASLD, and long COVID illustrate how names affect how conditions are understood, diagnosed, funded, researched, and experienced. Terminology should strive to reflect the science while minimising stigma and supporting equitable access to care; with patient perspectives prioritised throughout.

